# Practical Considerations of Workplace Wellbeing Management under Post-Pandemic Work-from-Home Conditions

**DOI:** 10.3390/ijerph21070924

**Published:** 2024-07-15

**Authors:** Victor K. L. Cheung

**Affiliations:** 1 Department of Neuroscience, Psychology & Behaviour, University of Leicester, Leicester LE1 7RH, UK; cheungklv.iop@gmail.com; 2 Multi-Disciplinary Simulation and Skills Centre, Queen Elizabeth Hospital, Hong Kong SAR, China

**Keywords:** COVID-19, teleworking, personnel management, occupational health, organizational innovation

## Abstract

As a natural experiment or “stress test” on the rapidly shifting work environment from office to home during and after the COVID-19 pandemic, staff wellbeing has been considered as the most critical issue in organizational change management. Following an overview of the relevant literature and recent official statistics, this essay aims to (i) address the major considerations and challenges in light of the transformation and re-design of the mode of work in the new normal and (ii) inform practical decisions for overall staff wellbeing under post-pandemic work-from-home (WFH) conditions with recommendations. For the sake of both staff healthiness and safety, as well as organizational competitiveness, senior management should take reasonable steps to enhance occupational safety in their WFH policy in line with practical recommendations on five areas, namely, (i) ergonomics, (ii) stress and anxiety management, (iii) workplace boundaries, (iv) work–family conflicts, and (v) other factors regarding a negative work atmosphere (e.g., loneliness attack, burnout, and workplace violence) particularly on virtual platforms. With the suggested evidence-based practices on WFH initiatives, senior management could make a difference in optimizing the overall workplace wellbeing of staff after the pandemic.

## 1. Introduction

### 1.1. Definition and Concept of Work from Home

The concept of work from home (WFH) originated from Nilles [1], using the terminologies “telework” and “telecommuting” to describe how employees perform duties in flexible workplaces wherever technology supports the communication and completion of tasks. WFH, interchangeable with remote work, e-working, home or virtual offices, became popular during the COVID-19 pandemic [2]. According to the Labor Force Survey, the proportion of WFH employees in the UK increased from 6% in 2017 to over 80% in the spring of 2020 [3]. In the United States, the proportion of WFH employees increased from 10% to 35% (or over 60% full-time employees), with over 70% of employees reporting a self-perceived work effectiveness [4].

Whether or not WFH is suitable as a mode of work for densely populated cities remains inconclusive [4]. Researchers found that the majority (80%) of employees in Hong Kong prefer WFH (either mixed or hybrid) to traditional office work as they appreciate more rest time (72%), lower work stress (>60%), less mental exhaustion (>80%), and flexible and compressed working hours (around 80%) [5]. With these appealing preliminary data, the following paragraphs will address the considerations of workplace wellbeing with theory and evidence in line with guidelines from BPS.

### 1.2. Psychological Models of WFH Arrangements

**Figure 1:**
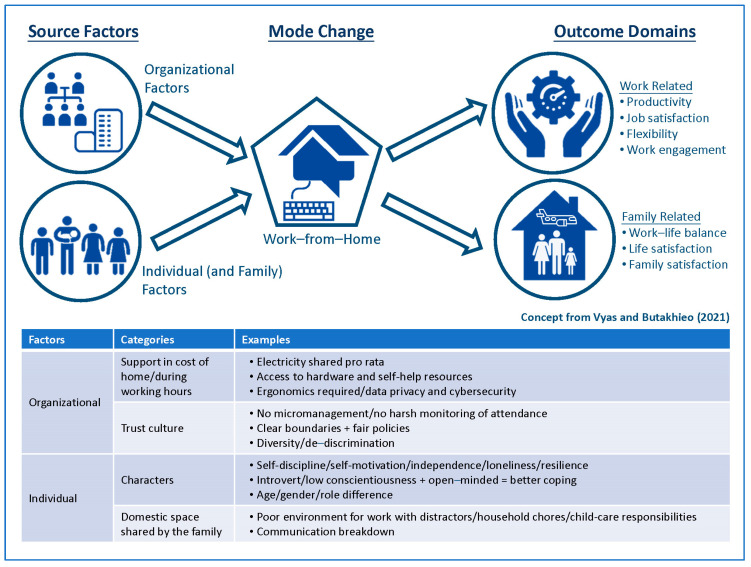
Analytic Framework of Work from Home during COVID-19 Pandemic.

An analytic framework has been established to inform structural considerations for a WFH arrangement and systemic effect on a variety of performance indicators during the pandemic [6]. In this model, “organizational” and “individual and family” factors determine outcomes on “occupation domain” (e.g., productivity, job satisfaction, flexibility, work engagement, and presenteeism/absenteeism) and “family domain” (e.g., work–life balance, life/family satisfaction, personal and family health conditions) (Figure 1).

With reference to guides on COVID-specific anxiety and distress in the workplace [7] and that on WFH conditions [8], the “SHARE” model has been disseminated as a psychologically informed approach for considerations towards a healthy and sustainable environment for WFH arrangements during the pandemic, namely, (i) safe homeworking, (ii) help yourself and others, (iii) adapt to change, (iv) relieve the pressure, and (v) evaluate (Table 1).

Due to the complicated relationship among multi-faceted elements, these analytic frameworks and guides help to structuralize key components for managing workplace wellbeing: occupation safety and health, stress and anxiety at work, boundaries, family conflict, and workplace conflict and violence.

## 2. Major Considerations

### 2.1. Occupational Safety and Health Consideration—Ergonomics

According to BPS [7,8], employers should have accountability for the workplace wellbeing of employees. Duty of care must include a risk assessment in the workplace, where ergonomics or human factors are key to workplace safety and health. The first practical challenge is ergonomically caused physical injury.

The Institute of Employment Studies [9] was commissioned during the COVID-19 lockdown to explore whether WFH arrangements would be good for employee wellbeing. Despite the coexisting effect of stress and anxiety induced by the lockdown or individual difficulties, non-standardized workplace settings triggered more complaints of musculoskeletal discomfort and unsatisfactory work–life balances from over half of the respondents. Gerding et al. [10] explored how ergonomics in home offices influenced the physical health of university staff members (*n* = 843). They found that compared with university office environments, participants had worked under suboptimal conditions (45% worked on a seat without adjustable armrests; 85% worked with a laptop instead of a desktop with a monitor and mouse). Despite reports including subjective feelings and having no track record for the actual onset date of physical discomfort, 40% of participants reported moderate-to-severe discomfort (e.g., severe low-back pain and moderate discomfort from head to shoulders) and worrying about developing musculoskeletal disorder in the long run.

Given the basic requirements of a workplace setup (e.g., the acquisition of technical skills, a computer, and an IT network, access to the necessary resources, and a connection to virtual teams), a non-standardized work environment, such as a sub-vented home with other co-tenants or one with too many distractors nearby, would pose a threat of cybersecurity or data privacy issues to employees and the organization. Managers should review the applicability of WFH arrangements with employees (e.g., the assessment of “Display Screen Equipment” covering all equipment during computer use), including the employees’ home conditions for occupational safety and health (OSH) purposes, enabling them to access the necessary technological support with cybersecurity devices and financially with additional devices (e.g., a work-specific computer and software and a chair with an armrest).

### 2.2. Stress and Anxiety at Work

Compared with family and other health-related issues, work has become a main source of stress in the general labor population [11]. The effects of chronic workplace stress regarding the wellbeing of staff, including excessive alcohol consumption, cardiovascular accidents, burnout, musculoskeletal disorders, overweight or obesity scores, eating and mood disorders, and mortality, have led to increased public awareness. This concerns its immense societal impacts on the overall productivity and costs of health insurance, as well as the healthcare burden on the global economy, which is over a trillion a year [12,13].

#### 2.2.1. Would WFH Conditions Lead to Positive Behavioral Change in Health Outcomes?

Researchers from Sweden explored the relationship of health conditions and behavioral change, as well as the distribution of time for sleep, work, leisure activity, and physical activity, by interviewing 27 full-time workers under WFH conditions [14]. Compared with the mode of office work, WFH arrangements resulted in an increased duration of sleep, largely saved from the non-office-hour work time (reduced OT). Quantity does not guarantee quality. At the other extreme, oversleeping would be detrimental to one’s physical and mental health [15]. Models addressing the degree to which sleeping time explains wellbeing outcomes and its association with physical activity and quality of life would be suggested for further studies during the post-pandemic phase.

#### 2.2.2. Do Personality Types Matter in WFH Conditions?

It is expected that people with a higher mood stability (or lower neuroticism) would cope better in WFH conditions because they were less likely pre-occupied with negative thoughts by working alone, which in turn can cause a psychological burden [11]. A common predictor for “excellent staff” in the workplace, conscientiousness, may be an interesting character under WFH conditions [16]. Based on about 200 sets of field data, Abbas and Raja [11] found that hindrance stressors were associated with psychological strains and turnover. In the face of the ever-evolving working environment during the pandemic, employees at a low end of conscientiousness (especially those with high openness) had lower turnover rates as high achievers because they tended to cope better with hindrance stressors (e.g., role ambiguity and overload) than their highly conscientious counterparts who stuck to deep-rooted mindsets in fixed and organized business landscapes [11]. The reasons for the conscientious employees leaving the organization were burnout, followed by overworking (with it being difficult to “switch-off”) and maladaptation as regards changes.

#### 2.2.3. Sense of Control and Job Satisfaction

In addition to the adverse health outcomes to get rid of, managers should take into account the positive aspects of workplace wellbeing, such as job satisfaction. Deci et al. [17] claimed that in order to foster job satisfaction, three senses, namely, autonomy, relatedness, and competence, are facilitators. Goh et al. [18] found that workplace wellbeing (e.g., physical and psychological health conditions and morbidity and mortality rates) was contributive to job control as regards the working pattern (e.g., flexi working hours and boundaries), self-autonomy (e.g., prioritization and workload), and power for decision making (communication, engagement, and teamwork), where the perceived fairness (with a constructive health policy in place) in the decision-making process and the interpersonal treatment throughout the course of the WFH period change the magnitude between them. A lack of control would likely consume more mental resources, decrease self-efficacy, and put employees at risk of burnout displayed by emotional exhaustion, depersonalization, and loss of personal accomplishment [19]. When job stability and flexibility were maintained in home working environments, the sense of security (e.g., daily engagement and finance source) and sense of control in each employee’s own pace of work may potentially trigger job satisfaction, higher productivity, less stress-induced anxiety, and conflict with family and peers [19,20,21]. The next paragraph will cover the control of boundary and work–family balance.

### 2.3. Workplace Boundaries

The negative influence of WFH arrangements has resulted in longer working hours, an isolation-induced sense of loneliness, and an invasion of “time and space” for families, as well as greater family influence on work [22]. Compared with home workers, office workers would have a relatively clear boundary: When I am off-duty, I leave the office physically. In the sense of “process of psychological detachment” or “switch-off”, physical attendance in an office has a symbolic meaning of “I am at work”, while leaving the office would be for “pastime”. WFH arrangements are actually breaking ground to make a physical and psychological separation between the work and office indifferent. Since the work–life boundary was blurred, Grant et al. [21] found that, under these conditions, employees tended to overwork during the pandemic, in particular those living with distractors (e.g., children under care) and high disengagement with colleagues [19,23].

To what extent and dimension should employers be accountable for employees’ wellbeing out of the office, in terms of fixed working hours, flexi-hours on an individual basis, or workload based on task output? Irrespectively, a clear boundary policy of the working hours and workload would be appreciated. Managers should also be aware of how excessive work demands affect not only the health condition of employees but also their relationship with family members.

### 2.4. Work–Family Conflicts

WFH arrangements would definitely put employees on a “stress test” under mixed conditions with both work–family interferences potentially being conflict-evoking. The classic scarcity hypothesis [24] indicated that personal resources, such as time, energy, and money, are limited and consumed under a zero-sum game: the more you invest certain resources into work, the less you can maintain a good relationship with your family. On the contrary, Solis [25] argued that, given fixed working schedules and individual spaces in home offices, time originally spent on traveling could be spent on building positive relationships with family members. Some scholars have examined relevant theoretical concepts by exploring how pressure from work triggers or intensifies inter-role conflicts with family members [26,27], categorizing work–family conflicts into different forms, namely, strain-based (negative emotions or pressure from one role affecting one’s fulfilment of another role), time-based (not meeting the obligations of the two roles due to time collisions) and behavior-based (the same patterns or characteristics not being compatible in two roles) across different magnitudes, such as family interference from work or work interference from family.

According to Giovanis et al. [2,28], common predictors in work–family conflicts include:

(i). Sex: Female WFH employees reported a positive life satisfaction and family relationship as the allocation of household chores and family responsibility was shared with their partners. Correspondingly, male employees’ slight decrease in job performance and productivity suggested a greater work influence from family than their partners.
(ii). Parental status: A positive association between the number of children the employee had and “work interference from family” were found. Married parents, especially those with a clear role delineation as breadwinner and houseworker, reported less inter-role conflicts than single parents, regardless of age and sex.
(iii). Other situational factors: Role stressors (role conflict, ambiguity, demands, and overload) showed a negative association with the role’s involvement in work–family conflicts, which implied a detachment from the role of work.
(iv). Which one has a greater impact on work–family outcomes, “family interference from work” or “work interference from family”? Regarding the indicators of satisfaction, job satisfaction is highly associated with “work interference from family”, while personal satisfaction (e.g., life or marriage) is highly associated with “family interference from work”.
(v). Psychological and physical strains are important indicators that link to the work–family relationship. For instance, burnout and alcoholism are more affected by a high level of work–family conflicts, particularly “work interference from family”, while a high-fat diet and physical inactivity were associated with “family interference from work”. Furthermore, work–family conflicts are attributive to poor sleep quality, amount of time spent asleep and nightmare frequency.

Irrespective of the direction of the interference on work–family conflicts, managers should provide the necessary measures to relieve pressure and evaluate its effects on employee wellbeing [7,8]. As the last resort, a psychological support service as well as an alternative WFH environment may be considered. 

### 2.5. Workplace Conflict and Violence in WFH Arrangements

WFH arrangements can be a double-edged sword to workplace harmony and destructive behavior in an organization. Arvola et al. [22] were optimistic about the positive influence of WFH conditions on autonomy (reducing direct confrontation in supervision), accountability, and professionalism, which would be extremely favorable to younger employees. However, Wech et al. [29] conducted a series of qualitative studies to prove that, except for the lack of communication, post-pandemic anxiety followed by excessive workloads and the change to WFH mode and process would increase cyberbullying and peer conflicts with passive-aggressive behavior as well as malfunctioned social competition.

Virtual platforms for WFH environments may not be easily adapted to by certain groups of employees, such as senior staff, those prone to digital fatigue or anxiety, and those with a physical disability. Such sudden changes in the work format without preparedness may trigger job insecurity or dissatisfaction or, even worse, exacerbate virtual team conflicts as well as workplace violence in digital form, such as online abuse/cyberbullying or spread of hate speech on inappropriate content (e.g., derogatory or obscene) [29].

Age discrimination following poor adaption to the specific technology required for WFH conditions is generally unprotected by law in most countries. Regarding seniority and specialty, additional support in advanced intercommunication and technology learning, good communication with complete information, and a decision-making process with the virtual teams could reduce the turnover rate of senior specialists and ensure productivity [30].

Affected by quarantine or lockdown under the pandemic, uncertainty and fear of being infected, anger for the restriction of outdoor activity, and the hatred of certain ethnic or minority groups have grown exponentially [2]. Cyberbullying, such as hostile and hate speech, and pointless accusations about certain racial groups spreading the virus may be the tip of the iceberg, particularly for those working with virtual teams comprised of members with diverse cultural background. A conflict resolution workshop and stress and emotion management, as well as a resilience workshop with a self-help toolkit, could be incorporated into any existing core competence training for internal staff on the virtual platform in order to dismantle discriminative or microaggressive behaviors under WFH conditions, such as gaslighting [30].

## 3. WFH-Specific Challenges: Social Contact—Loneliness and Wellbeing

The classic social comparison theory from Festinger [31] and the social learning theory from Bandura [32] asserted that individuals tended to reduce uncertainty by comparing the perception of others and themselves on certain statuses (e.g., sense of achievement) with internal feedback as an “objective benchmark” and be motivated to replicate behavior by observing peers with reciprocity and mutual goals. Beyond simply surviving as a human being, work can evoke a positive experience with the social contact of friendship, sense of identity, achievement on task completion, purpose or meaning of life fulfilment, and personal growth with resilience and character strengths when tackling a crisis [33,34]. Without face-to-face interaction under WFH conditions, employees would be attacked by loneliness in comparison with those having frequent direct interaction in the workplace.

As a result, the sense of loneliness or lack of socialization under isolation without timely feedback and mutual support from peers reduced self-efficacy and increased anxiety levels, which would result in overworking and burnout [2,35]. When people were urged to prove their value at work, they started to lose the work–life boundary in order to increase productivity, which might significantly reduce private time for their beloved ones (low social support) and taking a rest (low sleeping hours).

With 50% burnout rate in the global workforce [13], the real proportions and numbers may be underestimated because seriously burned-out employees would have remained unresponsive or quit their current full-time positions. From another angle, Collins et al. [36] argued that WFH arrangements provided staff who disliked their office environment with self-control on building positive relationships with those they want and avoiding conflicts with unfavorable peers. Nevertheless, the downsides of WFH conditions as regards team collaboration and communication with creativity and diversity remained non-demystified.

## 4. Power of Communication and Standardized Operation Guide

Ethically speaking, WFH arrangements should not be used by an organization for the sake of oppression. To strengthen WFH wellbeing as well as the productivity of the organization, communication with valid WFH policies, guidelines, and approaches is critical. McKinney and Company [13] indicated that WFH wellbeing (feeling supported and included) and productivity would show a two-to-five times increase when clear communication on relevant visions and policies has been achieved. Of over 5000 full-time employees, two-thirds were concerned about the existing communication breakdown in WFH approaches. When management supported in setting the boundary policy and team communication, as well as when the training of digitalization (on both technical and soft-skill-handling workload) was given, behavioral health and mental health conditions (e.g., depression, anxiety, and stress) of WFH employees were largely improved by 70% [12,14]. With the limitation of the large sample variation for synthesized findings, the inter-relatedness of the mentioned factors and the degree to which they could explain the overall wellbeing that followed WFH arrangements remained unknown.

## 5. Evidence-Backed Recommendation on Occupation Safety in WFH Arrangements

Actionable insights regarding WFH arrangements have been consolidated with practical recommendations applicable to occupational safety management.

### 5.1. Considerations in Ergonomics

To reduce the risk of acute physical injury (e.g., fracture from fall, neck sprain, etc.) and chronic health hazards (e.g., low back pain, musculoskeletal disorder, etc.), performing a preliminary assessment in determining the eligibility of the occupational safety of potential WFH environment is pivotal.

- In cases where the assessed environment was either suboptimal or impossible to improve with supplementary equipment offered by the company, a distanced-but-standardized workplace setting (such as a rental office or a library conference room with seats with adjustable armrests and an office notebook) would be much more suitable as opposed to a non-standardized workplace setting.
- Even if the assessed environment was considered largely eligible, managers are accountable for providing necessary operational, technical, and technological support to enable workers to execute their job duties under physically and psychologically safe conditions. They are also accountable for providing equipment and an environment assessment on a regular basis.

### 5.2. Stress and Anxiety at Work

To mitigate occupational stress- and anxiety-induced health hazards (e.g., sleep disturbance, being overweight, cardiac illness, alcoholism, etc.), reinforcing measures on healthy lifestyle modification, personality, and job satisfaction should be taken into account.

- With psychoeducation workshops implemented by healthcare professionals and psychologists who underwent qualified training in motivational interviewing techniques, it is anticipated that staff members under WFH arrangements could cope better with occupational stress and anxiety following changes in healthy lifestyle patterns, such as regular physical activity, cessation of excessive smoking, low alcohol consumption, and non-disruptive work and sleep quality with minimal doom-scrolling or midnight video conferences.
- Personality matters in workplace adaptation. Counterintuitively, staff members with a high conscientiousness might require extra guidance on WFH arrangements. The ever-changing nature of role ambiguity and overloading under unclear work boundaries took a massive toll on their physical and psychological health, leading to high burnout and turnover rates. Recapitulating the “switch-off” policy for working hours and active coaching on effective stress-coping techniques as well as time and workload management would be beneficial in reducing the burnout and turnover rates of valuable staff in the WFH mode.
- For positive aspects, ways to enhance job control and satisfaction guarantee the occupational wellbeing of staff and the sustainable development of the organization. Under a fair system of WFH conditions, managers could set the standard requirements (e.g., number of working hours, case load, formal meeting arrangements, recess intervals, etc.) and then allow flexibility for staff to maintain the sense of control by prioritizing their tasks, maintaining job satisfaction by being respectfully engaged in the decision-making process with optimal psychological safety.

### 5.3. Workplace Boundaries

Uncertainty of working boundaries, including time and space with family members and the sense of loneliness under a WFH status, must be handled with care.

- It is necessary for senior management to maintain a balance between setting boundaries (or clear switch-on and switch-off times) and providing ready support from supervisors and colleagues via a chosen electronic platform during official working hours.
- Asides from having a policy of standard working hours and workload in place, extra attention might be put towards the “overwork tendency” of those who have children to take care of in distanced workplaces and low engagement with peers. Supervisors may provide members in this target group with proactive mentoring on the progress of work and short-but-regular video conferences to strengthen the connectivity among team members on a weekly basis at minimum.

### 5.4. Work–Family Conflicts and Workplace Violence

It is essential for an organization to clearly define the sources of stress from the workplace and enrich their understanding of staff at all levels, informing staff about the related stress-reduction measures at primary, secondary, and tertiary intervention levels for stress management from a public health perspective.

- Special care should be given to those who have a higher proclivity to work–family conflicts (and lower job performance) under WFH conditions: (i) male, (ii) more children, (iii) unclear family role delineation if married, (iv) high role stressors at work, and (v) unhealthy lifestyle (e.g., poor sleep, alcoholism, physical inactivity, etc.).
- Regarding strain-based stress, managers might help staff to reduce role conflicts induced within the workplace or from their families by setting clear role-specific performance indicators in several phases. This could help staff to regain a level of control over demanding tasks towards feasible goals in the short term and long term.
- Regarding time-based stress, managers could reduce time collision by setting clear work boundaries and allowing flexibility for staff to complete tasks under reasonable timeframes based on personal schedules. Job redesign for specification and timeline may be required if regular reviews indicate that certain tasks are behind schedule owing to severe understaffing, high turnover rates, or absenteeism.
- Regarding behavior-based stress, managers may consider referring staff to a secondary intervention (e.g., relaxation, mindfulness or meditation group, psychosocial intervention training, or brief cognitive behavioral therapeutic sessions) managed by healthcare professionals. Individual fitness and an early stress-case-management program would be helpful in preventing the exacerbation of mild, unhealthy stress-coping patterns. In cases involving behavioral issues, whether highly complex or personal, immediate action regarding a preliminary workplace assessment and referral to a tertiary intervention (e.g., individual counseling or psychotherapy) should be taken in a timely manner. In addition to a psychological support service, an alternative WFH environment as opposed to a home environment after re-assessing the eligibility of the WFH arrangement may be arranged as the last resort.
- WFH-specific training with a live demonstration on how to activate off-site access to the database, work schedule, and communication platform and an enquiry hotline for technical, operational, and emotional support within office hours would be helpful in reducing any communication breakdown (e.g., excessive anxiety, maladaptive mode of work, and peer conflicts, etc.) and sense of loneliness. Establishing a robust WFH-specific workplace violence policy framework could build on the momentum of the culture change to dismantle cyberbullying at an organization-wide level.

With these suggested evidence-based practices for WFH initiatives, senior management could work together with all of the stakeholders to optimize sustainable growth in the wellbeing of their employees as well as in competitiveness of their organization after the pandemic.

## Figures and Tables

**Table 1:** SHARE Model from the British Psychological Society (BPS) [7,8].

Safe homeworking	Duty of care for staff safety Practical guide on roster, data privacy regulations, and equipment support Fairness/communication channels Ergonomics
Help yourself and others	Communicating and meeting needs ≥ set realistic targets Communication and check-ins/time-off regulations Technical/resilience skills Support hidden costs
Adapt to change	Diverse workplace situations and adjusting to the new normalcy for Diversity, Equity, and Inclusion (DEI) Personality/Individual differences Risk of cyberbullying/workplace violence Role conflict/work–life balance ≥ boundary setting
Relieve the pressure	Helping to adapt and cope with pressure from work and family
Evaluate	Reviewing the conditions regularly to ensure sustainable success (e.g., workspace and work format design/redesign)

## Data Availability

Not applicable.
